# Trends in Electronic Cigarette Use Among US Adults With a History of Cardiovascular Disease

**DOI:** 10.1001/jamanetworkopen.2023.28962

**Published:** 2023-08-15

**Authors:** Xin Wen, Tong Xia, Ruishan Li, Hongbin Qiu, Bo Yu, Yiying Zhang, Shanjie Wang

**Affiliations:** 1 Department of Epidemiology and Biostatistics, School of Public Health, Jiamusi University, Jiamusi, China; 2 Department of Cardiology, Second Affiliated Hospital of Harbin Medical University, Harbin, China; 3 The Key Laboratory of Myocardial Ischemia, Chinese Ministry of Education, Harbin, China

## Abstract

This cross-sectional study analyzes the prevalence of electronic cigarette (e-cigarette) use among adults with cardiovascular disease in the US between 2014 and 2020.

## Introduction

Tobacco use is a well-established behavior that substantially contributes to the increased disease burden associated with cardiovascular disease (CVD), leading to a considerable reduction in life expectancy of approximately 10 years.^1^ Since their introduction to the US market in 2006, electronic cigarettes (e-cigarettes) have gained notable popularity among smokers as a potential aid for smoking cessation.^2^ Several prior studies^3^ have reported e-cigarette prevalence and usage patterns in the US adult population. Notably, e-cigarette use has been found to be associated with increased adverse cardiovascular events.^4^ Thus, our cross-sectional study aimed to assess trends of e-cigarette use among adults with CVD to provide insight into the direction for future management of e-cigarette use.

## Methods

This study follows the (STROBE) reporting guideline and was exempt from institutional review board review and informed consent because the survey data were publicly available and deidentified, in accordance with 45 CFR §46. Participants were adults aged 18 years or older who responded between 2014 and 2020 to the National Health Interview Survey, an annual cross-sectional national health survey. Race and ethnicity were self-reported and included Asian, Black, White, and Hispanic ethnicity; and were analyzed to determine whether there were differences in e-cigarette use by race and ethnicity. We estimated the prevalence of self-reported current e-cigarette use along with 95% CIs, stratified by age, sex, and smoking status. Tests for trends were assessed using logistic regression considering year as a continuous variable. Multiple logistic regression was used to estimate the association of various factors with e-cigarette use. All statistical analyses were conducted using Stata statistical software version 15.1 (StataCorp). Statistical significance was considered with a 2-tailed *P* < .05. Data were analyzed from January 2023 to March 2023. Additional details are shown in the eMethods in Supplement 1.

## Results

**Figure 1.**
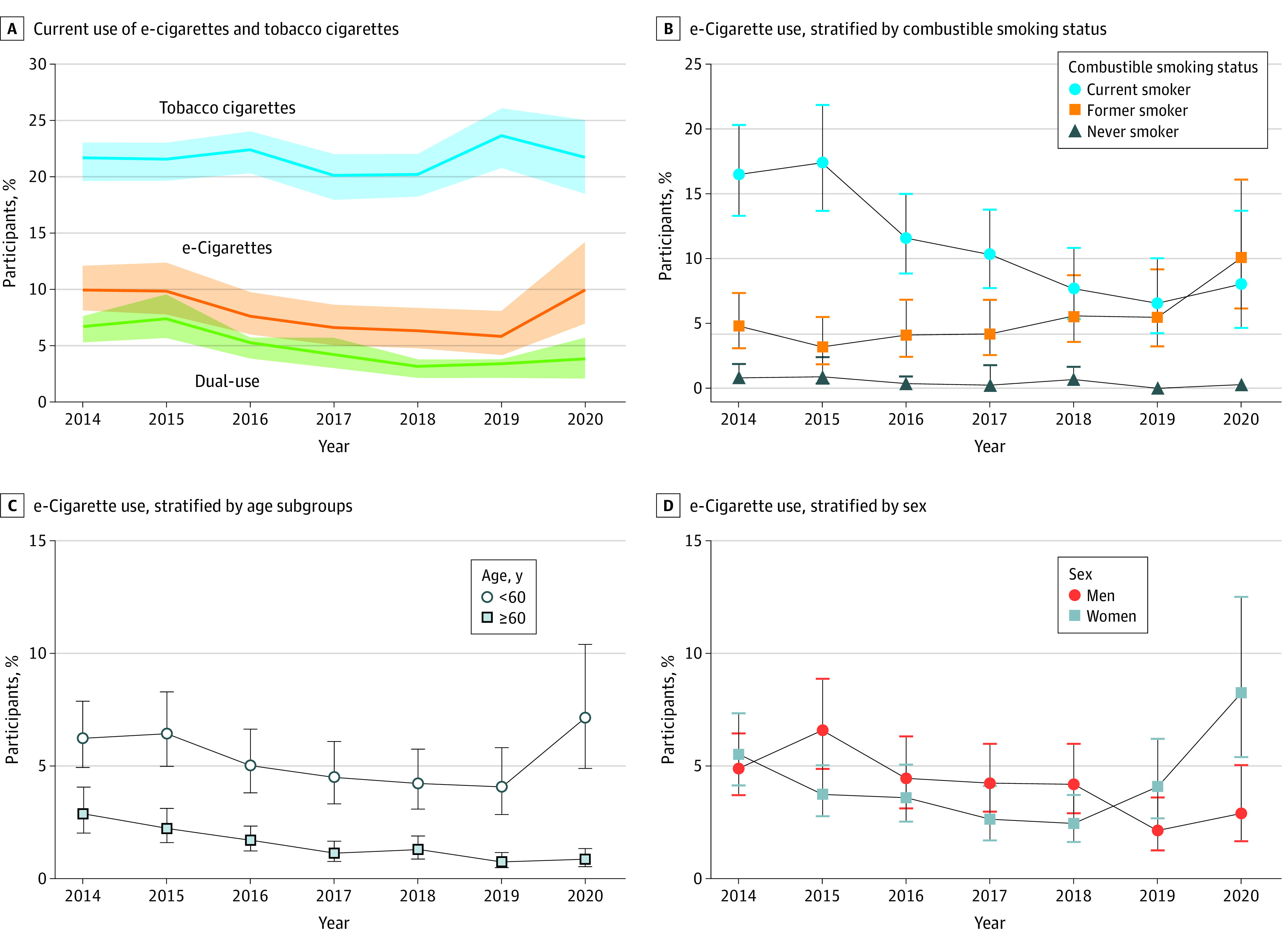
Trends of Tobacco Product Use Among US Adults With Cardiovascular Disease, 2014-2020 The figure shows overall weighted proportions of tobacco product use among US adults with cardiovascular disease (A) and subgroup analyses by patient smoking status (B), age (C), and sex (D). In panel A, solid lines represent adjusted prevalence estimates, and shaded areas represent the 95% CIs. In panels B, C, and D, solid shapes denote means and error bars denote 95% CIs. e-Cigarete indicates electronic cigarette.

**Figure 2.**
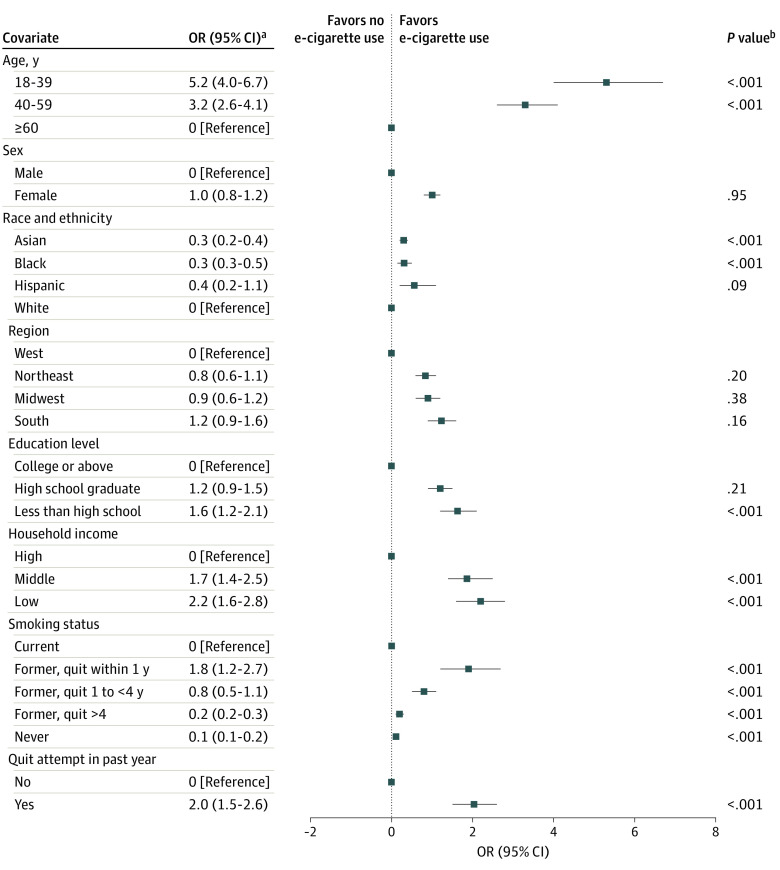
Factors Associated With Current Electronic Cigarette (e-Cigarette) Use Among US Adults with Cardiovascular Disease, 2014-2020 ^a^Odds ratios (ORs) and 95% CIs were calculated with multivariable logistic regressions adjusted for age, sex, region, race and ethnicity, education, and household income. ^b^Overall *P* value was calculated with the Wald test.

This study included 30 465 participants (mean [SD] age, 65 [15] years; 15 442 women [47.8%]; 1302 Asian participants [1.6%]; 3653 Black participants [4.9%]; 2443 Hispanic participants [4.9%] and 23 042 White participants [84.7%]). The weighted prevalence of current e-cigarette use decreased from 5.2% in 2014 to 3.1% in 2019 but rebounded subsequently to 5.2% in 2020 (Figure 1A). The prevalence of e-cigarette use among patients with CVD who quit smoking increased from 3.2% (34 users) in 2015 to 10.1% (32 users) in 2020 (Figure 1B). e-Cigarette use in patients aged 60 years or older decreased from 2.9% (73 users) in 2014 to 0.9% (24 users) in 2020, whereas use in those younger than 60 years remained consistently high (from 6.2% [128 users] in 2014 to 7.2% [34 users] 2020) (Figure 1C). Men were more likely to use e-cigarettes than women before 2018. However, the trend was reversed in 2019 and 2020 (2.9% [21 men] vs 8.3% [37 women] in 2020) (Figure 1D). Compared with current combustible smokers, those who had quit combustible tobacco (odds ratio, 1.8; 95% CI, 1.2-2.7) and those who tried but failed to quit tobacco within the past year (odds ratio, 2.0; 95% CI, 1.5-2.6) were more likely to use e-cigarettes (Figure 2).

## Discussion

In this cross-sectional study, e-cigarette use in patients with CVD decreased from 2015 to 2019, which may be partly due to the reported harms of e-cigarettes in recent years.^4^ However, the use of e-cigarettes rebounded in 2019, which may be explained by increased psychological burden during the COVID-19 pandemic.^5^ Consistent with reports in the general population,^3^ patients with CVD are also likely to use e-cigarettes as a smoking-cessation tool. e-Cigarettes may help control tobacco consumption,^2,4^ but whether the replacement of traditional cigarettes with e-cigarettes helps smokers improve cardiovascular health needs further verification. Given the reported cardiovascular hazards of e-cigarettes,^4^ smoking cessation, rather than e-cigarette substitution, may be more beneficial for cardiovascular secondary prevention. Notably, although smoking is traditionally thought to be a predominantly male activity, our study found that e-cigarettes are more popular among young individuals and women in recent years. Although our study was limited by self-reporting, e-cigarette use among patients with a history of CVD is noteworthy, especially among younger individuals and women and ex-smokers. Further studies are needed to understand the cardiovascular effects of e-cigarettes compared with combustible tobacco to inform future legislation for cardiovascular health.

## Notes

Supplement 1. **eMethods. **Study Population
eReference

Supplement 2.
Data Sharing Statement
