# Error in Discussion

**DOI:** 10.1001/jamanetworkopen.2023.34742

**Published:** 2023-09-12

In the Research Letter titled “Trends in Electronic Cigarette Use Among US Adults With a History of Cardiovascular Disease,”^1^ published August 15, 2023, there were errors in the years reported in the Discussion. These should have appeared as “…e-cigarette use in patients with CVD decreased from 2014 to 2019…” and “…the use of e-cigarettes rebounded in 2020…” This article has been corrected.^1^
