# Supplementary material for: Trends in Electronic Cigarette Use Among US Adults With a History of Cardiovascular Disease
Source: JAMA Netw Open. 2023 Aug 15;6(8):e2328962. doi: 10.1001/jamanetworkopen.2023.28962 (PMC10427939; doi:10.1001/jamanetworkopen.2023.28962)
Supplement: Supplement 1. — eMethods. Study Population eReference [file jamanetwopen-e2328962-s001.pdf]

Supplemental Online Content

Wen X, Xia T, Li R, et al. Trends in electronic cigarette use among US adults with a history of cardiovascular disease. *JAMA Netw Open*. 2023;6(8):e2328962.
doi:10.1001/jamanetworkopen.2023.28962

eMethods. Study Population

eReference

This supplemental material has been provided by the authors to give readers additional information about their work.

© 2023 Wen X et al. *JAMA Network Open.*

eMethods. Study Population

The current analysis included adults ages 18 years and older who reported a prior diagnosis of CVD, including stroke, coronary heart disease, angina pectoris, heart attack, or other heart disease, defined as any heart condition or disease.

Since 2014, participants were asked about their use of e-cigarettes.1 To establish e-cigarette use, the NHIS uses 2 questions: first, to establish ever use, the survey asks "Have you ever used an e-cigarette, even one time?" and second, to establish current use among those reporting ever use, "Do you now use e-cigarettes every day, some days, or not at all?" Those who responded "Yes" to the first question were categorized as ever e-cigarette users, and those that responded "every day" or "some days" to the second question were considered current e-cigarette users.

Covariates: age (18-39, 40-59, ≥60), sex (male, female), race/ethnicity (Asian, Black, Hispanic, White), geographic region (Northeast, Midwest, South, and West), education (above high school, high school, below high school), family income (in reference to the federal poverty limit from the Census Bureau, high income, >400% of federal poverty limit, middle income, 200%-400% of federal poverty limit, low income, <200% of federal poverty limit), smoking status (based on smoking history). Former smokers were categorized as those who had quit within the past 1 year, those who had quit between 1 and 4 years ago, and those who had quit 4 years or more ago.

eReference

1. National Center For Health S. National Center for Health Statistics, 2020.

© 2023 Wen X et al. *JAMA Network Open.*
