# Supplementary material for: Trends in Electronic Cigarette Use Among US Adults With a History of Cardiovascular Disease
Source: JAMA Netw Open. 2023 Aug 15;6(8):e2328962. doi: 10.1001/jamanetworkopen.2023.28962 (PMC10427939; doi:10.1001/jamanetworkopen.2023.28962)
Supplement: Supplement 2. — Data Sharing Statement [file jamanetwopen-e2328962-s002.pdf]

Data Sharing Statement

Wen. Trends in Electronic Cigarette Use Among US Adults With a History of Cardiovascular Disease. *JAMA Netw Open*. Published August 15, 2023.
doi:10.1001/jamanetworkopen.2023.28962

Data
Data available: Yes
Data types: Deidentified participant data
How to access data: https://www.cdc.gov/nchs/nhis/data-questionnaires-documentation.htm
When available: With publication

Supporting Documents
Document types: None

Additional Information
Who can access the data: anyone requesting the data
Types of analyses: for any purpose
Mechanisms of data availability: without investigator support
